# Automated local lockdowns for SARS-CoV-2 epidemic control—assessment of effect by controlled interrupted time series analysis

**DOI:** 10.1016/j.ijregi.2024.100380

**Published:** 2024-05-25

**Authors:** Laura Espenhain, Steen Ethelberg, Laust Hvas Mortensen, Lasse Engbo Christiansen

**Affiliations:** 1 Department of Infectious Disease epidemiology and Prevention, Statens Serum Institut, Copenhagen, Denmark; 2 Department of Public Health, University of Copenhagen, Copenhagen, Denmark; 3 Statistics Denmark, Copenhagen, Denmark; 4 Department of Epidemiology research, Statens Serum Institut, Copenhagen, Denmark

**Keywords:** Epidemiology, Containment strategies, Public health, SARS-CoV-2, Regional lockdown, Epidemic control

## Abstract

**Objectives:**

During the COVID-19 pandemic, broad non-pharmaceutical interventions such as national lockdowns were effective but had significant drawbacks, prompting targeted approaches, such as Denmark's localized lockdowns, based on specific epidemiological criteria. This study evaluates the effect of Denmark's automated local lockdown strategy on epidemic control to inform future response.

**Methods:**

This was a register-based controlled interrupted time series analysis, examining SARS-CoV-2 infection rates in Danish parishes from March to September 2021. The matching of control parishes was based on location, time, and pre-lockdown infection trends, with the lockdown's start defined as the day after a parish exceeded the lockdown criteria. Follow-up included 3-week pre-lockdown and 2-week post-lockdown.

**Results:**

A total of 30 parishes were mandated to lockdown, approximately 3.5% of the population of Denmark. A total of 94 control parishes were used as 109 controls. The decrease in the incidence during the 2-week follow-up period after the initiation of the lockdown was 13% points higher in case parishes: in case parishes, the incidence was reduced by 78% compared with 65% in control parishes.

**Conclusions:**

Our findings demonstrate that local lockdowns did have a positive effect in mitigating the spread of the SARS-CoV-2 virus, making them valuable in the fight against the COVID-19 pandemic and an important alternative to national lockdowns.

## Highlights

• Automated local lockdowns in Denmark were effectively containing virus transmission.
• Local lockdowns can be an important and less disruptive alternative to national lockdowns.
• Transparent and pre-set criteria may have played a positive role in garnering public compliance.

## Introduction

Non-pharmaceutical interventions (NPIs) were widely used in many countries during the COVID-19 pandemic. Reviews have found many NPIs to have been effective, in particular, those involving social distancing and lockdowns, although the effect of closing schools has been found less clear [1]. Because of the wide implication of imposing lockdowns of entire regions or countries, more targeted interventions are desirable. One such intervention policy was implemented in Denmark in the 2^nd^ year of the COVID-19 epidemic. This NPI targeted small geographical areas, which were required to go into lockdown if certain pre-set surveillance indicator thresholds were surpassed. An analysis of this potentially powerful NPI is the focus of this communication.

Parallel to a gradual relaxation of multiple national measures, a model for an “automated local lockdown” was adopted by the parliament and implemented from April 12, 2021. Although the majority of NPIs were implemented on national level, these automated local lockdowns were more localized interventions enacted by the 98 Danish municipalities in the 2142 (June 2021) Danish parishes. Parishes are the smallest administrative geographical unit in Denmark and are mainly (>98%) located within a single municipality. The automated local lockdown NPI required municipalities with parishes exceeding three epidemiological criteria set by the Danish Epidemic Committee to enact lockdown in the area. “Automated” refers to the system for identifying parishes that exceed the three lockdown criteria. The system was based on the Danish COVID-19 surveillance and every day at 2 pm, a list of all parishes and their performance on the three criteria was published online. The local lockdown included a switch to digital teaching and closure of after-school care and youth clubs in the parish. The local lockdown further required municipalities to close venues and locations where cultural activities were taking place, as well as venues where sports, leisure, or other activities were being held in the parish. Municipalities could resume activity in a parish if one of the epidemiological criteria had been below the threshold 7 days in a row.

Benefitting from these local lockdowns in Denmark, we aim to investigate the effect of such lockdowns on the progress of the epidemic in the area to inform future measures and preparedness plans to reduce the spread of infection.

## Material and methods

### Study design

The study was a register-based controlled interrupted time series analysis of SARS-CoV-2 infections in Danish parishes. The study was based on national SARS-CoV-2 polymerase chain reaction (PCR) test result data from the Danish COVID-19 surveillance system. The study period was from March 23 to September 14, 2021, covering the period when the automated local lockdowns were mandatory and allowing 3 weeks of observation pre-lockdown and 2 weeks of follow-up post-lockdown. All residents in Denmark are registered in the national population register, with demographic information, including place of residence.

### Setting

The Danish response to the COVID-19 pandemic included free and easily accessible testing, independent of medical referral [2], [3]. During the study period, several daily activities required residents in Denmark to have a recent negative SARS-CoV-2 test, a registered previous infection, or proof of vaccination. This resulted in a very high test activity of around 20,000 weekly tests per 100,000 population during the first part of the study period and around 7000-10,000 weekly tests per 100,000 population from June 2021. Free-of-charge COVID-19 vaccination was being rolled out nationally during the study period. Roll out was by vulnerable population groups and by descending age. By mid-April 2021, 80% of those above 75 years of age had received the first vaccine. By mid-September (end of study period), 88% of the population above 20 years of age had received two doses.

### Data source, populations, and follow-up

We used data from the Danish COVID-19 surveillance system, which includes data on all reverse transcription-PCR SARS-CoV-2 tests, test results, and residence (parish) of the tested person, automatically captured through register data. From the daily aggregated surveillance data, we identified all parishes exceeding the lockdown criteria (i.e. case parishes) during the study period. We included parishes exceeding the lockdown criteria for more than 1 day. To account for potential place-varying confounding, control parishes were selected from parishes close to the case parish (within a 30-km radius of). To account for potential time-varying confounding, we matched on time by comparing the same period (the 5 weeks from 21 days before until 14 days after initiation of the local lockdown) in case and control parishes. We matched on the pre-lockdown SARS-CoV-2 trend to account for the natural dynamics of outbreaks. We sorted potential control parishes according to how close the incidence of SARS-CoV-2 the week before lockdown was to that of their case parish and selected up to five controls for each case parish. Case parishes could serve as control parishes during periods outside their own 5-week observation period pre- and post-lockdown.

The 1^st^ day of the local lockdown was defined as the day after the announcement that the case parish exceeded the lockdown criteria. For the interrupted time series analysis, we defined two periods: a 3-week pre-lockdown period and a 2-week post-lockdown period. The pre-lockdown period ended (t = 0) the day before local lockdown (i.e. the day of the announcement), after which the post-lockdown period started, ending 2 weeks after the lockdown had been implemented (t = 2).

### Study outcomes and epidemiological measures

The primary outcome was the 7-day cumulated number of people with a positive SARS-CoV-2 PCR test per 100,000 persons in a parish. We used the 7-day cumulated incidence as opposed to daily to limit bias from varying test activity throughout the week.

To explore any potential change in behavior pre-lockdown based on fear of a potential near-future lockdown, we described the timing of the peak number of SARS-CoV-2 cases in relation to the day of the initiation of the local lockdown in case and control parishes. We defined the peak as the day during the 5-week follow-up period with the highest 7-day cumulated number of people with a positive SARS-CoV-2 test. If the peak number of cases occurred on more than 1 day, we used the latest date.

### Statistical analyses

We compared the shift in trends in the 7-day cumulated SARS-CoV-2 incidence in the 2 weeks after the initiation of the local lockdown in the general population of case parishes with that of matched control parishes (match parish [0;1]). We used generalized linear regression in a mixed-effect model, specifically the glmer() function from the lme4-package in R version 4.3.1, RStudio v. 2023.06.2, using Poisson with the logarithm of the population within the parish as the offset. We allowed random intercepts and pre- and post-lockdown slopes (weeks relative to lockdown [−2 to 2] and weeks since lockdown [0-2]) for each match group (case parish and up to five control parishes) and random intercepts for each parish.

Our model included a parameter for shift in trend (match parish × weeks since lockdown) and did not include a parameter to allow an immediate effect of the lockdown. If not available directly from model output, we extracted the 95% confidence intervals (CIs) from the model using bootMer and fixef in the lme4-package.

The effect associated with the lockdown during the 2-week period after initiation of the local lockdown was visualized by applying the effect (slope) seen in control parishes to case parishes. The lockdown criteria changed three times during the study period (Table 1); we stratified the analyses on the four periods (April 12-April 29, April 30-May 27, May 28-July 15, and July 16-August 31). To test whether the shift in trend depended on the incidence at initiation of lockdown, we included an interaction term between an index of how close a parish was to the incidence criteria on the day before the lockdown and time (weeks) since the lockdown was initiated. Because the interaction term was insignificant, we did not keep it in the final model.

**Table 1:** Timeline of thresholds of the three criteria prompting local lockdowns.

Periode	Past 7 days in parish^a^			Number of parishes starting lockdown during the period
(2021)	Number of SARS-CoV-2 infections		Percent positive tests	
	Per 100,000	absolute number		
April 12-29	400	≥20	2%	14
April 30-May 27	500	≥20	2.5%	7
May 28-July 15	600	≥20	3%	6
July 16-August 31	1,000	≥20	3%	5

a 2-9 days.

We present the model in which the lockdown parish is reference. We also ran a similar model in which the control parish was the reference and used the 95% CI for the interaction term to describe the effect of the lockdown.

### Sensitivity analyses

To shed light on any “spillover”/scare effect in parishes in close vicinity versus natural progression of propagated outbreaks, we also did the controlled interrupted time series analysis using parishes 30-70 km from the case parish as controls and thus not affected by the local epidemic situation in or around case parishes.

To shed light on the specific effect of school closures which were part of the bundle measures, we stratified the original controlled time series analyses on whether there was a school in the parish or not.

### Ethics

For this study, we have used publicly available aggregated register data. According to Danish law, ethical approval is not to be obtained for register-based studies not using biological material. The study was approved by the legal department at Statens Serum Institut, according to regulations by the Danish Data Protection Agency.

## Results

**Figure 1:**
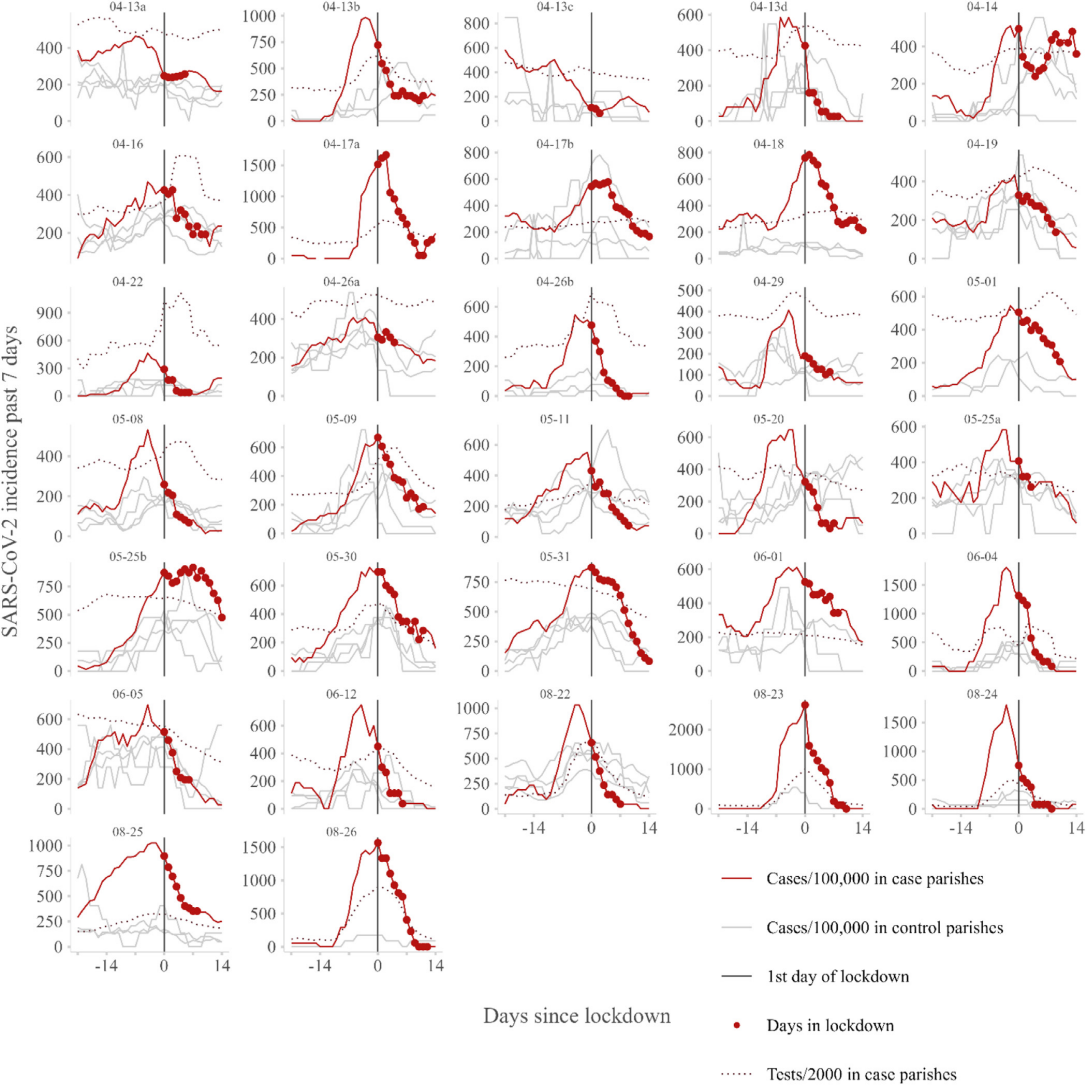
Timeline for the 32 case parishes: daily 7-day cumulated incidence of SARS-CoV-2 per 100,000 population in case (red line) and control (gray lines) parishes and daily cumulated 7-day number of tests per 2000 population in case parishes (red dotted line) in the period from 21 days before to 14 days after the day of lockdown, sorted by date of lockdown (seen in upper left corner of each graph. Letters after the dates indicate that more than one parish started lockdown on the given date). The vertical full line marks the 1^st^ day of lockdown and the red dots marks the actual days a parish exceeded the lockdown criteria.

During the study period, a total of 30 parishes in 20 municipalities were mandated to lockdown. The first parishes closed on April 13 and the last on August 26, 2021 (Figure 1). The duration of the exeedance of the lockdown criteria ranged from 3 to 24 days, with a median of 9.5 days. Three parishes experienced two lockdowns during the study period. For one of these parishes, there was only 1 day between the two exceedances and only the first lockdown was included in the analysis. Consequently, a total of 32 local lockdowns were included in the study. A total of 14 parishes closed during the first period with the strictest criteria (Table 1). The population of the case parishes varied from 1066 to 22,191 individuals, with a median population of 6049. In total, the case parishes accounted for 193,544 individuals, which is approximately 3.5% of the population in Denmark.

A total of 94 control parishes were selected and used as 109 controls. The majority of case parishes had five matched control parishes, whereas six parishes had 1-4 controls and no matched controls could be found for one case parish. For each case parish, the daily 7-day cumulated incidence of SARS-CoV-2, days in lockdown, and number of SARS-CoV-2 tests per 2000 population can be seen in Figure 1; in addition, the daily 7-day cumulated incidences of SARS-CoV-2 in control parishes are plotted. The observed peak incidence in case parishes ranged from 406 to 2627 SARS-CoV-2 per 100,000. The daily number of SARS-CoV-2 tests of the previous 7 days per 2000 population ranged from 39 to 1120 (median 356, interquartile range [IQR] 271-472) in case parishes and 41-962 (median 371, IQR 284-457) in control parishes during the study period. The daily 7-day cumulated incidence of SARS-CoV-2 peaked and turned, on average, 2.5 days before lockdown in case parishes (median −3, IQR −4 to −0.75) and 2 days before lockdown in control parishes (median −1, IQR −5 to 2).

### Effect of automated local lockdown

**Figure 2:**
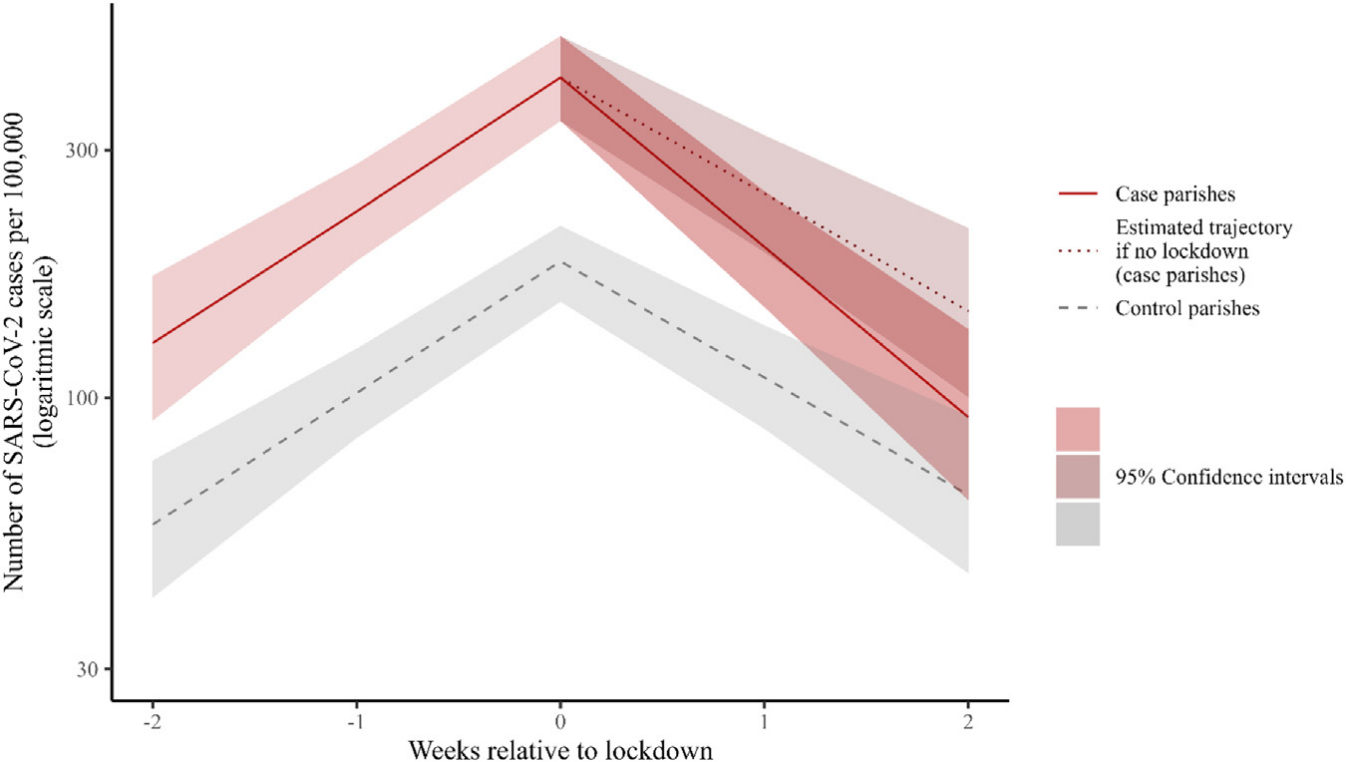
Modeled 7-day cumulated incidence of SARS-CoV-2 per 100,000 in week −2 to week 2 relative to the local lockdown in parishes which were in lockdown (red lines), including the predicted trajectory if they had not been in lockdown (red dashed line) and control parishes (gray dashed lines) during period 1 (April 12-April 29, 2021).

Based on the model estimates (Table 2), the incidence of SARS-CoV-2 in control parishes the week preceding lockdown was 45% (CI 38-52%) of that in case parishes (e.g. estimated 332-502/100,000 in case parishes vs 151-219/100,000 in control parishes during period 1, visualized on Figure 2). The incidence of SARS-CoV-2 the week preceding lockdown was 90% (incidence rate ratio [IRR] = 1.904 95% CI 1.377-2.640, Table 2) higher in period 3 than in period 1. In the pre-lockdown period, incidence increased 81% each week (IRR = 1.805, 95% CI 1.535-2.147, Table 2) in case and control parishes.

**Table 2:** Incidence rate ratios of predictors (fixed effects), random effects and characteristics of the controlled time series model.

Predictors	Incidence rate ratio	95% confidence interval	*P*-value
Intercept	0.004	0.003 - 0.005	<0.001
Control parish	0.446	0.383 - 0.519	<0.001
Weeks relative to lockdown	1.805	1.536 - 2.122	<0.001
Weeks since lockdown	0.261	0.201 - 0.340	<0.001
April 30-May 27	1.380	1.054 - 1.806	0.019
May 28-July 15	1.904	1.390 - 2.609	<0.001
July 16-August 31	1.907	1.352 - 2.690	0.001
Control parish x weeks since lockdown	1.263	1.181 - 1.349	<0.001
Random effects			
σ²	6.24		
τ00 match_group:sogn_f	0.11		
τ00 match_group	0.06		
τ11 match_group:weeks_rel_lockdown	0.19		
τ11 match_group:t_w_since_1d	0.49		
ρ01 match_group:weeks_rel_lockdown	0.44		
ρ01 match_group:t_w_since_1d	-0.22		
ICC	0.07		
N match_group	31		
N sogn_f	122		
Observations	720		
Marginal R² / Conditional R²	0.052/0.122

During the 2 weeks after the initiation of the local lockdown, a declining trend was observed in case and control parishes (visualized for period 1 in Figure 2). In case parishes, the slope indicated a halving of the number of SARS-CoV-2 cases per week after lockdown (IRR 1.805 × 0.261 = 0.47, 95% CI 0.40-0.55), which was 15-26% faster than the decline observed in control parishes. In a supplementary analysis, we included test activity in the model and found similar patterns (Supplementary Table 1). In control parishes, the post-lockdown trend corresponded to a 31-49% reduction of the number of cases per week (IRR = 1.805 × 0.261 × 1.262 = 0.595) in the main analysis.

The decrease in the incidence during the 2-week follow-up period after the initiation of the lockdown was 13% points (8-19% points) higher in case parishes than in control parishes: in case parishes, the incidence was reduced by 78% (95% CI 70-84) compared with 65% (95% CI 52-74%) in control parishes. The predicted trajectory of case parishes, had they had the same trajectory of control parishes, is also visualized in a red dashed line (Figure 2).

### Sensitivity analyses

When using controls not in close vicinity to the case parishes, the estimated effect (slope change) from the local lockdown did not change (7.6-20% vs 15-26%). The daily 7-day cumulated incidence of SARS-CoV-2 peaked and turned, on average, 0.8 days before lockdown in control parishes (median 1 day before lockdown, IQR: −3.25 to 2.0).

Among the case parishes, 24 had between 1-8 primary or lower secondary schools located in them, whereas six had no schools in this category. When stratifying the original controlled time series analyses by whether there was a school in the locked down parish or not, the estimated effect of the lockdown was a 13-24% faster decrease in incidence in parishes with a school (n=26) and a 24-52% faster decrease in incidence in parishes without schools (n=6).

## Discussion

The automated local lockdown of parishes was implemented as a response to the SARS-CoV-2 pandemic to contain the spread of infection and protect public health. In this study, we aimed to investigate the effect of these local lockdowns on the number of cases in the affected areas. Our findings demonstrate that the local lockdown measure did indeed have a positive effect in mitigating the spread of the SARS-CoV-2 virus. Comparing parishes which had been in lockdown to control parishes, we estimated a 13%-point decrease in SARS-CoV-2 incidence during the 2 weeks after the initiation of the lockdown. Interestingly, the daily 7-day cumulated number of cases declined already a few days before the implementation of the lockdown in the case parishes and the control parishes. Finally, we found that relatively few parishes ended up in lockdown; thus, less than 4% of the population of Denmark were directly affected by these automated lockdowns.

A pre-intervention effect has been described by Berry et al. [4] and Goolsbee et al. [5] in their studies on shelter-in-place policies and mobility policies, and Goolsbee et al. [5] note that individual choices were far more important and seem tied to fears of infection. One could expect a direct effect of the intervention after at least one incubation period, i.e. 4-5 days, after implementing the intervention. In our case, the SARS-CoV-2 incidence peaked and turned already before the lockdown was initiated, including in the control parishes. This suggests that factors other than the specific intervention might have contributed to the decline in cases during the period, potentially indicating a selection-history interaction. It also highlights the importance of having a control group for analyses as these. Because local politicians and the local population could follow how their own—and e.g. neighboring—parishes performed on the lockdown criteria on a daily basis, the pre-lockdown effect in case parishes could be due to behavioral changes, as such, the incidence began to approach the threshold described in the lockdown criteria. This behavioral change could include more testing, less social interaction, and mobility. Because the incidence in the control parishes were less than half of that in case parishes—far from the incidence threshold—such an effect may not have been as marked here. However, neighboring parishes, e.g. in the same municipality, and individuals attending school, culture, or sport activities in a parish which was approaching the lockdown threshold may also have changed behavior despite not living there. Chernozhukov et al. [6] have shown that people voluntarily reduced their visits to workplace, retail stores, and grocery stores when there was a higher number of new cases and deaths. Nevertheless, because this effect was also observed in control parishes not in close vicinity to the case parish, as described in the sensitivity analysis, natural progression of local outbreaks where a marked peak is followed by a decline back to “normal” may also explain part of the observed effect in control parishes.

The lockdown resulted in a faster decline in the number of SARS-CoV-2 cases in the parish than in control parishes, equivalent to 54-103 spared SARS-CoV-2 cases per 100,000 during the 2 weeks after, depending on the period, or a total number of SARS-CoV-2 cases of around 150 spared during the 5-month period. Any long-term effects, e.g. changed behavior, after the 14-day follow-up period, or spillover effects to neighboring parishes are obviously not captured in this figure. Because only three parishes closed more than one time, such an effect may have been in place.

In April 2021, when the system for automated local lockdowns was implemented, several other national NPIs were in place, alongside vaccination efforts [7]. These included the “Corona passport,” which provided access to public spaces for individuals with proof of vaccination, previous infection, or a negative SARS-CoV-2 test [7]. In late April, restaurants, cafés and bars, and shopping malls, reopened with restrictions. Face masks were mandatory in various settings. Employees in the public sector were mandated to work from home if their job function allowed it, with a gradual easing during the study period to full capacity from August 1, 2021.

The model for automated local lockdowns included closure of primary and secondary schools, after-school care and clubs, and universities. National restrictions in schools were in place during parts of the study period, April and May, with 50% in-person learning for pupils in grades 5-8 in lower secondary schools and in upper secondary schools, as well as 20% for most university students [7]. Bi-weekly testing for in-person learning in individuals 12 years or older was in place during the whole study period. In addition, the model for automated local lockdowns included closure of venues and places where cultural activities, sports, leisure, or other activities were taking place. Cultural institutions required a valid Corona passport for entry, and national gathering restrictions for public spaces were in place. For outdoor sports, the gathering restriction was lifted by the end of May and replaced with a requirement of a valid Corona passport [7]. From our study, it is not possible to separate the effect of the specific measures in the lockdown bundle. Concerning the closure of educational institutions, most parishes did not have upper secondary schools or universities but did have primary and lower secondary schools affected by the lockdowns. Several previous studies have explored the specific effect of school closures during the COVID-19 pandemic using interrupted time series designs [8], [9], [10] and positive effects have been reported [8], [9], [10], although Rotevatn et al*.* [8] in Oslo, Norway found that school closures were equally effective as targeted infection prevention and control measures in open schools in reducing student infection rates. Similarly, other study designs, such as an event-study/difference-in-differences approach, have been used to evaluate the effects of reopening schools [11], [12], reporting effects on the population level [11], [12] to small and transitory effects only in certain subgroups [13]. Concerning the closure of cultural, sport, or leisure intuitions/venues, Barbeito et al. in Spain have attempted to estimate the specific effects of various NPIs, including sports and culture activities, but their results for NPIs targeting culture, leisure venues, and indoor sports were inconsistent. They found no effect of closing outdoor sport venues in decreasing transmission [14].

Few studies have focused on the effect of local lockdowns on SARS-CoV-2 incidence. Interestingly, two modelling studies from the pre-vaccination period did not find local lockdowns in the UK or France to be sufficient to prevent outbreak rebound [15], [16]. Our aim was not to evaluate the relative effect of local compared with national lockdowns nor the effect or the specific measures included in the bundle; we find that the local lockdowns did have a positive effect on reducing the SARS-CoV-2 incidence in the area and note that other concurrent NPIs were relaxed during the study period, indicating that the measures in places were found sufficient to keep the epidemic at an acceptable level.

Although our study provides valuable insights into the effects of local lockdowns, its limitations should be acknowledged. We conducted an observational register-based study, which inherently introduces certain limitations. We designed the study to accommodate these in the best way possible. A key strength of our design was the inclusion of a control group, enhancing the internal validity of our findings. The inclusion of the control group changed the effect estimate vastly (52% vs 12% decrease each week). History bias due to other interventions or events occurring around the time of the intervention is the primary threat to the validity of interrupted time series studies [17]. We matched on time and geography to account for potential time- and place-varying confounding factors, such as seasonality, vaccination coverage, dominant SARS-CoV-2 variant, and other NPIs in place to reduce the spread of infection. To explore potential effects from a changed test-activity pattern, we included test activity in the analysis and found similar effect of the local lockdown intervention.

The automated local lockdowns were guided by clear and national criteria and proved to be an effective strategy in reducing the transmission of the virus within the limited affected areas, whereas the epidemic situation remained stable in the rest of the country. The presence of transparent and pre-set criteria may have played a positive role in garnering public compliance. However, the fact that parish borders did not overlap with school districts presented a communication challenge. Nevertheless, the local anchoring of these measures within the communities may have fostered a sense of shared responsibility and empowerment among politicians and residents. As we navigate the challenges of future pandemics, it is crucial to address remaining unanswered questions. We find that the local automated lockdowns implemented in Denmark were effective and constitutes a valuable and less invasive instrument for future pandemic control, which is also argued by others [18]. Determining the optimal criteria and geographical unit of local lockdowns requires further investigation.

## Conclusion

Considering the significant disruptions and substantial negative impact on society caused by lockdowns, including school closures, it is imperative to limit their use to only situations where it is absolutely needed. Our study demonstrates that during a period with Alpha and Delta SARS-CoV-2 variants, local lockdowns were effective in curbing the spread of SARS-CoV-2 in the areas, making them valuable in the fight against the COVID-19 pandemic and, very importantly, a crucial alternative to national lockdowns.

## Declarations of competing interest

The authors declare the following financial interests/personal relationships which may be considered as potential competing interests:

Laura Espenhain have received a PhD-grant from the Independent Research Fund Denmark (10.46540/2061-00050B) to conduct this research. The other authors declare no competing financial interests or personal relationships that could have appeared to influence the work reported in this paper.

## Appendix Supplementary materials

Image, application 1
