# Supplementary material for: Automated local lockdowns for SARS-CoV-2 epidemic control—assessment of effect by controlled interrupted time series analysis
Source: IJID Reg. 2024 May 25;12:100380. doi: 10.1016/j.ijregi.2024.100380 (PMC11186853; doi:10.1016/j.ijregi.2024.100380)
Supplement: Supplementary file 1 [file mmc1.pdf]

Supplementary content to

Automated local lockdowns for SARS-CoV-2 epidemic control – assessment of effect by controlled interrupted time series analysis

By

Laura Espenhain, MSc[a]

Steen Ethelberg, professor[a,b]

Laust Hvas Mortensen, professor [c,b]

Lasse Engbo Christiansen, senior researcher[d]

Affiliations:

[a] Department of Infectious Disease epidemiology and Prevention, Statens Serum Institut,

Copenhagen, Denmark

[b] Department of Public Health, University of Copenhagen, Copenhagen, Denmark

[c] Statistics Denmark, Copenhagen, Denmark

[d] Epidemiology research, Statens Serum Institut, Copenhagen, Denmark

This supplementary material has been provided by the authors to give readers additional information about their work

Original statistical methods

We compared the shift in trends in the 7-day cumulated SARS-CoV-2 incidence in the two weeks following the initiation of the local lockdown in the general population of case parishes with that of matched control parishes [match parish (0;1)]. We used generalized linear regression in a mixed-effect model, specifically the glmer() function from the lme4-package in R version 4.3.1, RStudio v. 2023.06.2 using Poisson with the logarithm of the population within the parish as the offset. We allowed for random intercepts and pre- and post-lockdown slopes [Weeks relative to lockdown (-2 - 2) and Weeks since lockdown (0 - 2)] for each match group (case parish and up to five control parishes) and random intercepts for each parish.

Our model included a parameter for shift in trend [Match parish x Weeks since lockdown] and did not include a parameter to allow for an immediate effect of the lockdown. If not available directly from model output, we extracted the 95% confidence intervals from the model using bootMer and fixef in the lme4-package.

The effect associated with lockdown during the 2-week period after initiation of the local lockdown, was visualized by applying the effect (slope) seen in control parishes to case parishes. The lockdown criteria changed three times during the study period (Table 1), we stratified the analyses on the four periods [April 12 – April 29, April 30 – May 27, May 28 – July 15, July 16 – August 31]. To test whether the shift in trend depended on the incidence at initiation of lockdown we included an interaction term between an index of how close a parish was to the incidence criteria on the day prior to lockdown and time (weeks) since lockdown was initiated. As the interaction term was insignificant, we did not keep it in the final model.

We present the model in which the lockdown parish is reference. We also ran a similar model in which the control parish was the reference and used the 95%CI for the interaction term to describe the effect of the lockdown.

Additional supplementary methods

To explore potential effects from a changed test-activity pattern, we included test activity in the analysis and found similar effect of the local lockdown intervention.

```
final_model_incl_log_tests <- glmer(N_cases7d ~ status + weeks_rel_lockdown + t_w_since_ld +
period + log(N_tests7d/pop) +  status:t_w_since_ld +
                                   (1 + weeks_rel_lockdown + t_w_since_ld | match_group) +
                                   (1 | match_group:sogn_f)
                                   + offset(log(pop)),
                                       family = poisson, data = data,
                                       control = glmerControl(optimizer = c("bobyqa"))
                                       )
```

Additional supplementary results

Supplementary Table 1: Incidence rate ratios of predictors (fixed effects), random effects and characteristics of the controlled time series model including test activity

Predictors	Incidence rate ratio	95%CI	p-value
Intercept	0.011	0.008 - 0.015	<0.001
Control parish	0.476	0.407 - 0.556	<0.001
Weeks relative to lockdown	1.620	1.420 - 1.848	<0.001
Weeks since lockdown	0.328	0.266 - 0.405	<0.001
April 30-May 27	1.319	1.013 - 1.717	0.040
May 28-July 15	1.853	1.382 - 2.487	<0.001
July 16-August 31	2.407	1.741 - 3.329	<0.001
log(tests/population)	1.934	1.647 - 2.270	<0.001
Control parish x Weeks since lockdown	1.232	1.152 - 1.317	<0.001
**Random Effects**			
σ^2	6.14		
τ00 match_group:sogn_f	0.12		
τ00 match_group	0.04		
τ11 match_group:weeks_rel_lockdown	0.12		
τ11 match_group:t_w_since_1d	0.27		
ρ01 match_group:weeks_rel_lockdown	0.21		
ρ01 match_group:t_w_since_1d	0.00		
ICC	0.06		
N match_group	31		
N sogn_f	122		
Observations	720		
Marginal R^2 / Conditional R^2	0.051/0.108		
